# Person-centered health education and training

**DOI:** 10.3325/cmj.2014.55.79

**Published:** 2014-02

**Authors:** Marijana Braš, Veljko Đorđević

**Affiliations:** Centre for Palliative Medicine, Medical Ethics and Communication Skills, School of Medicine University of Zagreb, Zagreb, Croatia

## The model of Person-Centered Medicine and the First International Congress of the International College of Person-Centered Medicine

During the last decade, a series of new models have transformed our understanding of the concept of health and of the implementation of health care systems. Person-centered medicine (PCM) model is one of them (1). The International College of Person-Centered Medicine (ICPCM) promotes medicine of the person, for the person, by the person, and with the person. The ICPCM emerged from the ongoing annual Geneva Conferences as a strong international network of dozens of leading world-wide medical organizations and institutions (2-4). The first Geneva Conference on Person-Centered Medicine was held in May 2008, under the auspices of ten global institutions, including medical organizations, international bodies of health professionals, and associations of patients and families. Starting with the Fifth Geneva Conference, a *Geneva Declaration* was regularly issued, focusing on the conference’s main theme and was widely distributed (5,6). The First International Congress of the ICPCM was held in Zagreb, Croatia on November 7-10, 2013. The main theme was the Whole Person in Health Education and Training. Zagreb was chosen due to the enduring legacy of professor Andrija Štampar, president of the first WHO World Health Assembly, founder of Zagreb University’s School of Public Health, and pioneer of person- and people-centered care. Gathering participants from 29 countries, the Congress was a true example of an interdisciplinary collaboration. The emphasis was on establishing strong links between experts dealing with science, clinical work, and art in medicine, as well as between experts and patient groups (and patients themselves along with their families). Students also had an important role and contemporary teaching methods were promoted, eg, the use of trained actors as simulated patients, and patients as teachers. Also the integration of all fields of health care when working with patients and their families was emphasized, which is a relationship which must truly be a collaborative partnership. As health care in general is a very complex and dynamic system, reflecting social changes, in recent decades great attention has been paid to the quality of communication in medicine and medical interview (7-9). When we talk about different forms of communication in medicine, we must never forget the importance of communication through art (10). Person-centered management takes into account psychological, physical, social, and spiritual aspects of health and disease. Therefore, education in medicine must be interdisciplinary and directed toward the person rather than the symptom, diagnosis, or disease, with the emphasis on experiential learning. In a community, whether local, national, or international, we must work together toward the same goal: a culture of person-centered medicine and public-centered health care.

## Zagreb Declaration and Zagreb Statement

Declarations are relevant to the framing of crucial areas in health care, raising awareness, and facilitating transfer of knowledge across the different stakeholders involved in health care. During the Zagreb Congress, two documents were formulated and adopted: the Zagreb Declaration on Person-Centered Health Professional Education (11) and the Zagreb Statement on the Appraisal and Prospects for Person-Centered Medicine in Croatia (12). The Zagreb Declaration on Person-centered Health Professional Education consists of eight recommendations. In the Zagreb Statement on the Appraisal and Prospects for Person-Centered Medicine in Croatia, formulated by local participants and the local organizing group, it was emphasized that activities in Croatia can contribute significantly to the development of person-centered medicine and people-centered health care, in Croatia and abroad.

Person-centered medical education is a key priority in the implementation of person-centered medicine and people-centered health care. We must see patients as persons within their physical, psychological, social, and spiritual totality. *Ars medica* (the art of medicine) is both art in medicine and medicine of art, as well as a journey from the culture of illness toward the culture of health, from symptoms and diagnoses toward man and person. Medical education is a critical component of person-centered health care, which is developed from the ground up and requires a revised approach to teaching of clinical skills (among them, communication skills). It is the first step in the establishment of a culture of person-centered medicine among its practitioners and patients alike.

## References

[1] Mezzich JE, Salloum IM, Cloninger CR, Salvador-Carulla L, Kirmayer LJ, Banzato CE (2010). Person-centred integrative diagnosis: conceptual bases and structural model.. Can J Psychiatry.

[2] Mezzich JE (2011). The Geneva Conferences and the emergence of the International Network of Person-centered Medicine.. J Eval Clin Pract.

[3] Mezzich JE (2011). Building person-centered medicine through dialogue and partnerships: perspective from the international network for person-centered medicine.. Int J Pers Cent Med..

[4] Mezzich JE, Snaedal J, van Weel C, Botbol M, Salloum I, van Lerberghe W (2011). Articulating Person-centered Medicine and People-centered Public Health: A Report from the Fourth Geneva Conference.. World Med J.

[5] Miles A, Mezzich JE (2012). Person-centered medicine: addressing chronic illness and promoting future health.. International Journal of Person Centered Medicine..

[6] Mezzich JE, Appleyard J (2013). Continuing development of person centered medicine and addressing chronic disease.. International Journal of Person Centered Medicine..

[7] Đorđević V, Braš M, Miličić D. Introduction. In: Đorđević V, Braš M, Miličić D. Person in medicine and healthcare: from bench to bedside to community. Zagreb: Medicinska naklada; 2012. p. 43.

[8] Đorđević V, Braš M, Brajković L (2012). Person-centered medical interview.. Croat Med J.

[9] Đorđević V, Braš M, Milunović V, Brajković L, Stevanović R (2011). The founding of the Centre for Palliative Medicine, Medical Ethics and Communication Skills: a new step toward the development of patient oriented medicine in Croatia.. Croat Med J.

[10] Braš M, Đorđević V, Janjanin M (2013). Person-centered pain management – science and art.. Croat Med J.

[11] (2014). Zagreb Declaration on Person-Centered Health Professional Education. Croat Med J.

[12] (2014). Zagreb Statement on the Appraisal and Prospects for Person-Centered Medicine in Croatia. Croat Med J.

